# Re-Visiting *Trichuris trichiura* Intensity Thresholds Based on Anemia during Pregnancy

**DOI:** 10.1371/journal.pntd.0001783

**Published:** 2012-09-13

**Authors:** Theresa W. Gyorkos, Nicolas L. Gilbert, Renée Larocque, Martín Casapía, Antonio Montresor

**Affiliations:** 1 Parasite Epidemiology Research Group, Division of Clinical Epidemiology, Research Institute of the McGill University Health Centre, Montreal, Canada; 2 Department of Epidemiology, Biostatistics and Occupational Health, McGill University, Montreal, Canada; 3 International Development Research Centre, Ottawa, Canada; 4 Asociación Civil Selva Amazónica, Iquitos, Perú; 5 Department of Control of Neglected Tropical Diseases, World Health Organization, Geneva, Switzerland; Case Western Reserve University School of Medicine, United States of America

## Abstract

**Background:**

The intensity categories, or thresholds, currently used for *Trichuris trichiura* (ie. epg intensities of 1–999 (light); 1,000–9,999 epg (moderate), and ≥10,000 epg (heavy)) were developed in the 1980s, when there were little epidemiological data available on dose-response relationships. This study was undertaken to determine a threshold for *T. trichiura*-associated anemia in pregnant women and to describe the implications of this threshold in terms of the need for primary prevention and chemotherapeutic interventions.

**Methodology/Principal Findings:**

In Iquitos, Peru, 935 pregnant women were tested for *T. trichiura* infection in their second trimester of pregnancy; were given daily iron supplements throughout their pregnancy; and had their blood hemoglobin levels measured in their third trimester of pregnancy. Women in the highest two *T. trichiura* intensity quintiles (601–1632 epg and ≥1633 epg) had significantly lower mean hemoglobin concentrations than the lowest quintile (0–24 epg). They also had a statistically significantly higher risk of anemia, with adjusted odds ratios of 1.67 (95% CI: 1.02, 2.62) and 1.73 (95% CI: 1.09, 2.74), respectively.

**Conclusions/Significance:**

This analysis provides support for categorizing a *T. trichiura* infection ≥1,000 epg as ‘moderate’, as currently defined by the World Health Organization. Because this ‘moderate’ level of *T. trichiura* infection was found to be a significant risk factor for anemia in pregnant women, the intensity of *Trichuris* infection deemed to cause or aggravate anemia should no longer be restricted to the ‘heavy’ intensity category. It should now include both ‘heavy’ and ‘moderate’ intensities of *Trichuris* infection. Evidence-based deworming strategies targeting pregnant women or populations where anemia is of concern should be updated accordingly.

## Author Summary

Infection by the soil-transmitted helminth *Trichuris trichiura* is defined as ‘light’, ‘moderate’ and ‘heavy’ depending on its intensity. However, these intensity categories were developed in the 1980s, before any epidemiological data were available on the association between specific *T. trichiura* infection intensities and adverse health outcomes. Here, we re-analyzed data from a study of *T. trichiura* infection and anemia in pregnant women to determine the threshold (i.e. the lowest infection intensity) associated with an increased risk of anemia. Women with *T. trichiura* infections of intensities ranging from 601 to 1632 eggs per gram of feces (epg) (ie. a ‘moderate’ level of intensity) had a significantly higher prevalence of anemia and a significantly lower hemoglobin level than the reference group (i.e. women with *T. trichiura* infections of intensities ranging between 0 and 24 epg). This finding contrasts with the common belief that only ‘heavy’ *T. trichiura* infection (10,000 epg and above) can cause anemia.

## Introduction

The most recent comprehensive estimation of the prevalences of the soil-transmitted helminthiases (STH) documents a global prevalence of 17% for *Trichuris trichiura* infection, with approximately 800 million persons infected at any one time [1], [2]. Community-wide prevalences are frequently over 30–40% and it is not uncommon to observe prevalences exceeding 80% in community sub-groups like school-age children and preschool-age children [3]–[7]. *T. trichiura* infections contribute to the STH-attributable burden of disease by adversely affecting the growth and cognitive development of children and the health and productivity of adults [8], [9]. Because of its co-occurrence with other infections, malnutrition and poverty, it also diminishes the economic potential, not only of the infected individual, but also of the family and community as well [10].

In 1987, an expert committee convened by the World Health organization (WHO) established infection intensity categories for STH, including *T. trichiura*, in order to inform the management of large-scale deworming programs [11]. *T. trichiura* infection was defined as light (1–999 epg) or heavy (>10,000 epg) [11]. These categories were based primarily on expert opinion and little dose-response data from the field, and were described as “arbitrary” by this committee. [11]. A further category of ‘moderate’ (i.e. for epg counts between 1,000 and 9,999 epg) was subsequently added by WHO [12]. The original 1987 report had also mentioned that anemia attributable to *T. trichiura* infection reflected a ‘very heavy worm burden’ [11].

Since then, the association between *T. trichiura* (prevalence and intensity) and hemoglobin (Hb) levels or anemia, has been assessed in several epidemiologic studies mostly conducted in Africa and in Asia and of which the majority found no significant association [13]–[18]. However, four studies conducted in the Americas (Jamaica, Panama, Mexico and Peru) reported statistically significant associations [19]–[22]. In addition, *T. trichiura* infection has been associated with a lower increase of Hb in iron-supplemented pregnant women [22]. Mechanisms by which *T. trichiura* infection may cause anemia include ingestion of blood by the parasite, blood loss from parasite-induced lesions in the intestinal mucosa, and inflammatory responses such as tumor necrosis factor α (TNFα) leading to decreased appetite; the relative contributions of these factors being unknown [9].

Anemia is a major public health problem because it impairs the growth and cognitive development in children and because severe anemia increases the risk of maternal mortality. Its worldwide prevalence is estimated at 48.8% [23]. The importance of the cluster of STH to the global risk of anemia is relatively well known, but among helminth species, *T. trichiura* has received much less attention than hookworms.

The objectives of this study were to determine a threshold for *T. trichiura*-associated anemia in pregnant women, and to describe the implications of this threshold in terms of the need for primary prevention and chemotherapeutic interventions.

## Methods

### Ethics Statement

Ethics approval was obtained for the original RCT from the following review committees: Research Institute of the McGill University Health Centre (Canada), The “Comite Institucional de Etica de la Universidad Peruana Cayetano Heredia” (Peru); and the “Comite Etica de la Direccion General de Salud de las personas del Ministerio de Salud de Peru” (Peru). The research procedures followed were in accordance with the ethical standards of these three ethics committees and with the Helsinki Declaration. Written informed consent was obtained from all women.

The data source for this study originated from a randomized controlled trial on mebendazole during pregnancy and its effect on birth weight which had been conducted in the highly STH-endemic Amazon area of Peru whose methods have been described elsewhere [24]. Briefly, 1,042 pregnant women were recruited in their second trimester and randomly assigned to receive either a single dose of 500 mg mebendazole or a placebo. Women in both groups received daily iron supplements throughout their pregnancy. At enrolment (second trimester) and again in the third trimester, blood and stool specimens were collected from participants for hemoglobin (Hb) ascertainment by HemoCue and for STH determination by the Kato-Katz method. There was no statistically significant difference between intervention groups in the prevalence of anemia or in mean hemoglobin levels in the third trimester. However, women having *Trichuris trichiura* infection in the second trimester were at a higher risk of anemia in their third trimester [22].

To determine a threshold for the effect of *T. trichiura* infection intensity on hemoglobin and anemia, the 935 mothers for whom complete information was available (i.e. on helminth infection and hemoglobin level in both the 2nd and 3rd trimester, plus covariates) were divided into quintiles based on *T. trichiura* infection intensity in the second trimester. Mean hemoglobin concentrations and anemia prevalence in the third trimester were calculated for each group. Mean hemoglobin concentrations in the third trimester of each *T. trichiura* quintile were compared to the lowest quintile using generalized linear model (GLM) analysis. The prevalence of anemia, defined as hemoglobin <11 g/dL [23], in the third trimester in each quintile was compared to that of the lowest quintile by logistic regression. Covariates found to be statistically significantly associated with the outcome were included in regression models: the model predicting hemoglobin levels included hookworm intensity and the model predicting anemia included hookworm intensity and the time interval between assessments for hemoglobin levels [22].

## Results

Among the 935 pregnant women included in the analysis, 82% were infected with *Trichuris trichiura*, and 43% were co-infected with *T. trichiura* and hookworms. The highest *T. trichiura* infection intensity was 25,200 epg. Participants' characteristics are described in more detail elsewhere [22].

Women in the lowest three *T. trichiura* intensity quintiles had similar hemoglobin concentrations, with arithmetic mean levels of 11.53, 11.55 and 11.58 g/dL, respectively. In contrast, the fourth and fifth quintiles had significantly lower mean hemoglobin concentrations than the reference group (i.e. 11.24 and 11.05 g/dL, respectively) (Table 1). The fourth and fifth quintiles also had a statistically significantly higher risk of anemia, with adjusted odds ratios of 1.67 (95% CI 1.02, 2.62) and 1.73 (95% CI 1.09, 2.74), respectively (Table 2).

**Table 1:** Association between *Trichuris* infection in the second trimester and hemoglobin levels in the third trimester.

*Trichuris*	n	Hemoglobin (g/dL)		Simple regression			Multiple regression^1^		
(epg)		mean	SD	β	SD	p	β	SD	p
0–24	189	11.53	1.02	0.00			0.00		
25–240	188	11.55	1.18	0.02	0.11	0.844	0.05	0.12	0.688
241–600	191	11.58	1.10	0.05	0.11	0.664	0.09	0.12	0.424
601–1632	180	11.24	1.14	−0.29	0.12	0.013	−0.24	0.12	0.046
≥1633	187	11.05	1.09	−0.48	0.11	<0.001	−0.38	0.12	0.002

1 Adjusted for hookworm infection intensity.

**Table 2:** Association between *Trichuris* intensity in the second trimester and anemia risk in the third trimester.

*Trichuris* intensity (epg)	N	n anaemic	% anaemic	Crude OR	95% CI			Adjusted OR^1^	95% CI		
0–24	189	54	28.57	1.00				1.00			
25–240	188	64	34.04	1.29	0.83	,	2.00	1.24	0.80	,	1.93
241–600	191	50	26.18	0.89	0.57	,	1.39	0.84	0.53	,	1.34
601–1632	180	75	41.67	1.79	1.16	,	2.75	1.67	1.07	,	2.62
≥1633	187	81	43.32	1.91	1.25	,	2.93	1.73	1.09	,	2.74

1 Adjusted for hookworm infection intensity and for the time interval between the first and second assessments.

## Discussion

The fact that a statistically significant association between *T. trichiura* infection and anemia was found in this study, but not in any other study of pregnant women, can be explained, in part, by the fact that this time the association between *T. trichiura* and anemia was determined in a population of women who had received daily iron supplements. Therefore, the fraction of anemia attributable to an insufficient dietary intake may have been reduced in the study population, resulting in an increased fraction attributable to *T. trichiura*. This likely strengthened the association between *T. trichiura* and anemia in our study population, a finding that may not have been easily observable in other populations.

The 601–1632 epg *T. trichiura* infection intensity category was the lowest epg category where a statistically significant association between hemoglobin and anemia was found. This indicates that the threshold for the *T. trichiura* effect on hemoglobin and the risk of anemia in iron-supplemented pregnant women appears to be somewhere between 601 and 1632 epg. In other words, iron-supplemented pregnant women with “light” or “moderate” *T. trichiura* infection intensities, based on the current classifications, may indeed be at an increased risk of morbidity from anemia as a result of the infection.

This finding has implications for STH control programs, in particular, those programs targeting pregnant women, because the efficacy of the commonly used deworming regimens of single-dose albendazole or mebendazole against *T. trichiura* is not optimal [25].

This analysis also provides support for categorizing a *T. trichiura* infection ≥1,000 epg as “moderate”, as currently defined by WHO. In addition, for pregnant populations, even if they are receiving iron supplements during pregnancy, it may be that 601 epg should be considered a lower limit for this ‘moderate’ category.

The most important implication of these analyses is that moderate *T. trichiura* infection in pregnant women is a significant risk factor for anemia which, in turn, increases the risk of adverse maternal and infant health outcomes. Therefore, in a pregnant population where there is a high prevalence of *T. trichiura* infection and where intensity levels exceed 600 epg, it may be that additional care options beyond the commonly used single-dose albendazole or mebendazole should be considered.

## References

[1] Albonico M, Allen H, Chitsulo L, Engels D, Gabrielli AF, et al (2008) Controlling soil-transmitted helminthiasis in pre-school-age children through preventive chemotherapy. PLoS Negl Trop Dis 2: e126. 18365031 10.1371/journal.pntd.0000126 PMC2274864

[2] de Silva NR, Brooker S, Hotez PJ, Montresor A, Engels D, et al (2003) Soil-transmitted helminth infections: Updating the global picture. Trends Parasitol 19: 547–551. 14642761 10.1016/j.pt.2003.10.002

[3] Albonico M, De Carneri I, Di Matteo L, Ghiglietti R, Toscano P, et al (1993) Intestinal parasitic infections of urban and rural children on Pemba Island: Implications for control. Ann Trop Med Parasitol 76: 579–583. 10.1080/00034983.1993.11812813 8122919

[4] Forrester JE, Bailar JC, Esrey SA, José MV, Castillejos BT, et al (1998) Randomised trial of albendazole and pyrantel in symptomless trichuriasis in children. Lancet 352: 1103–1108. 9798586 10.1016/s0140-6736(97)08325-6

[5] Needham C, Kim HT, Hoa NV, Cong LD, Michael E, et al (1998) Epidemiology of soil-transmitted nematode infections in Ha Nam Province, Vietnam. Trop Med Int Health 3: 904–912. 9855404 10.1046/j.1365-3156.1998.00324.x

[6] Rahman WA (1998) Helminthic infections of urban and rural schoolchildren in Penang Island, Malaysia: implications for control. Southeast Asian J Trop Med Public Health 29: 596–598. 10437964

[7] Taylor M, Pillai G, Kvalsvig JD (1995) Targeted chemotherapy for parasite infestations in rural black preschool children. S Afr Med J 85: 870–874. 8545746

[8] Crompton DWT, Nesheim MC (2002) Nutritional impact of intestinal helminthiasis during the human life cycle. Annu Rev Nutr 22: 35–59. 12055337 10.1146/annurev.nutr.22.120501.134539

[9] Stephenson LS, Holland CV, Cooper ES (2000) The public health significance of *Trichuris trichiura* . Parasitology 121 Supplement:S73–S95. 11386693 10.1017/s0031182000006867

[10] Hotez PJ, Fenwick A, Savioli L, Molyneux DH (2009) Rescuing the bottom billion through control of neglected tropical diseases. Lancet 373: 1570–1575. 19410718 10.1016/S0140-6736(09)60233-6

[11] World Health Organization (1987) Prevention and Control of Intestinal Parasitic Iinfections. Report of a WHO Rxpert Comittee. Technical Report Series 749. Geneva: World Health Organization. 3111104

[12] Montresor A, Crompton DWT, Hall A, Bundy DAP, Savioli L (1998) Guidelines for the Evaluation of Soil-Transmitted Helminthiasis and Schistosomiasis at Community Level. Geneva: World Health Organization.

[13] Olsen A, Magnussen P, Ouma JH, Andreassen J, Friis H (1998) The contribution of hookworm and other parasitic infections to hemoglobin and iron status among children and adults in western Kenya. Trans R Soc Trop Med Hyg 92: 643–649. 10326110 10.1016/s0035-9203(98)90795-7

[14] Brooker S, Peshu N, Warn PA, Mosobo M, Guyatt HL, et al (1999) The epidemiology of hookworm infection and its contribution to anemia among pre-school children on the Kenyan coast. Trans R Soc Trop Med Hyg 93: 240–246. 10492749 10.1016/s0035-9203(99)90007-x

[15] Ezeamama AE, McGarvey ST, Acosta LP, Zierler S, Manalo DL, et al (2008) The synergistic effect of concomitant schistosomiasis, hookworm, and Trichuris infections on children's anemia burden. PLoS Negl Trop Dis 2: e245. 18523547 10.1371/journal.pntd.0000245 PMC2390851

[16] Ndyomugyenyi R, Kabatereine N, Olsen A, Magnussen P (2008) Malaria and hookworm infections in relation to hemoglobin and serum ferritin levels in pregnancy in Masindi district, western Uganda. Trans R Soc Trop Med Hyg 102: 130–136. 17996912 10.1016/j.trstmh.2007.09.015

[17] Kung'u JK, Goodman D, Haji HJ, Ramsan M, Wright VJ, et al (2009) Early helminth infections are inversely related to anemia, malnutrition, and malaria and are not associated with inflammation in 6- to 23-month-old Zanzibari children. Am J Trop Med Hyg 81: 1062–1070. 19996438 10.4269/ajtmh.2009.09-0091

[18] Midzi N, Mtapuri-Zinyowera S, Mapingure MP, Sangweme D, Chirehwa MT, et al (2010) Consequences of polyparasitism on anemia among primary school children in Zimbabwe. Acta Trop 115: 103–111. 20175980 10.1016/j.actatropica.2010.02.010

[19] Robertson LJ, Crompton DWT, Sanjur D, Nesheim MC (1992) Hemoglobin concentrations and concomitant infections of hookworm and *Trichuris trichiura* in Panamanian primary schoolchildren. Trans R Soc Trop Med Hyg 86: 654–656. 1287935 10.1016/0035-9203(92)90176-d

[20] Ramdath DD, Simeon DT, Wong MS, Grantham-McGregor SM (1995) Iron status of schoolchildren with varying intensities of *Trichuris trichiura* infection. Parasitology 110: 347–351. 7724242 10.1017/s0031182000080938

[21] Quihui-Cota L, Morales-Figueroa GG, Esparza-Romero J, Valencia ME, Astiazarán-García H, et al (2010) Trichuriasis and low-iron status in schoolchildren from Northwest Mmexico. Eur J Clin Nutr 64: 1108–1115. 20700138 10.1038/ejcn.2010.146

[22] Gyorkos TW, Gilbert NL, Larocque R, Casapía M (2011) *Trichuris* and hookworm infections associated with anemia during pregnancy. Trop Med Int Health 16: 531–537. 21281406 10.1111/j.1365-3156.2011.02727.x

[23] de Benoist B, McLean E, Egli I, Cogswell M (2008) Worldwide Prevalence of Anemia 1993–2005. WHO Global Database on Anemia. Geneva: World Health Organization.

[24] Larocque R, Casapía M, Gotuzzo E, MacLean JD, Soto JC, et al (2006) A double-blind randomized controlled trial of antenatal mebendazole to reduce low birthweight in a hookworm-endemic area of Peru. Trop Med Int Health 11: 1485–1495. 17002722 10.1111/j.1365-3156.2006.01706.x

[25] Keiser J, Utzinger J (2008) Efficacy of current drugs against soil-transmitted helminth infections: Systematic review and meta-analysis. JAMA 299: 1937–1948. 18430913 10.1001/jama.299.16.1937

